# The Involvement of the Omentum in Defensive Peritoneal Mechanisms: New Evidence from Dogs Naturally Infected with *Leishmania infantum*

**DOI:** 10.3390/ani16152336

**Published:** 2026-07-31

**Authors:** Hélio Noberto de Araújo, Telma de Sousa Lima, Moacir Franco de Oliveira, Radan Elvis Matias de Oliveira, Francisco Antônio Félix Xavier, Tiago Cunha Ferreira, Daniel de Araújo Viana, Diana Célia Sousa Nunes-Pinheiro

**Affiliations:** 1 Centro de Educação, Ciências e Tecnologia da Região dos Inhamuns, Universidade Estadual do Ceará, Tauá 63660-000, CE, Brazil; telma.lima@uece.br (T.d.S.L.); daniel.viana@uece.br (D.d.A.V.); 2 Programa de Pós-Graduação em Ciências Veterinárias, Universidade Estadual do Ceará, Fortaleza 60714-903, CE, Brazil; tiago.cunha@uece.br (T.C.F.); diana.pinheiro@uece.br (D.C.S.N.-P.); 3 Programa de Pós-Graduação em Biotecnologia, Universidade Estadual do Ceará, Fortaleza 60714-903, CE, Brazil; 4 Programa de Pós-Graduação em Ciência Animal, Universidade Federal Rural do Semi-Árido, Mossoró 59625-900, RN, Brazil; moacir@ufersa.edu.br (M.F.d.O.); radan.oliveira@ufersa.edu.br (R.E.M.d.O.)

**Keywords:** canine leishmaniosis, immune cells, immunopathogenesis, omental tissue, peritoneal immunity, systemic inflammatory biomarkers

## Abstract

The omentum is an important immune surveillance organ located in the abdominal cavity, especially noteworthy in diseases displaying tropism for the mononuclear phagocyte system, such as canine leishmaniosis (CanL). In this context, this study aimed to evaluate the immune-inflammatory response of the omentum in dogs naturally infected with *Leishmania infantum* through systemic inflammatory biomarkers and anatomopathological alterations. A total of 40 dogs were investigated, comprising 10 seronegative animals and 30 symptomatic seropositive dogs with CanL. Following clinical and laboratory evaluations, median celiotomy was performed and omental samples sent to histopathological analyses. Infected dogs displayed normocytic normochromic anemia, dysproteinemia, hypoalbuminemia, hyperglobulinemia, significant reductions in albumin-to-globulin ratios (AGR), and increased neutrophil-to-albumin ratios (NAR), with these as the main inflammatory biomarkers. Microscopically, chronic inflammatory infiltrates presenting a predominant lymphoplasmacytic pattern were observed, associated with hyperemia, fibrosis, and granulomas, in addition to the presence of amastigote *Leishmania* spp. forms within the macrophage cytoplasm. A strong association was observed between histological inflammation grade and inflammatory pattern, whereas erythrogram parameters showed progressive reductions with increasing inflammatory grades. The omentum is, thus, noted as actively participating in CanL immunopathogenesis, acting as a potential niche for parasite persistence and a complementary indicator of systemic disease severity, while AGR and NAR demonstrate potential diagnostic and prognostic value for the clinical monitoring of canine leishmaniosis.

## Simple Summary

Canine leishmaniosis is an important disease that affects dogs and is of major public health relevance, since these animals serve as the primary urban reservoir for leishmaniosis. Although the disease is usually studied in organs such as the spleen, liver, and bone marrow, little is known about the role of the omentum, a fatty tissue located inside the abdomen that helps protect organs and participates in the body’s immune defense. This study investigated changes in the omentum of dogs naturally infected with the leishmaniosis parasite and their association with blood inflammatory markers. Infected dogs presented anemia, blood protein alterations, and signs of chronic inflammation. Inflammation, fibrosis, and chronic lesions known as granulomas were observed in the omentum, while parasite forms were found within macrophages, supporting the involvement of this tissue. More severe omental damage was associated with more pronounced blood changes, especially anemia. These findings demonstrate that the omentum may act as an important site for parasite persistence and disease progression. In addition, blood markers such as albumin and neutrophil-related changes may aid veterinarians in improving the diagnosis, prognosis, and clinical monitoring of affected dogs.

## 1. Introduction

The omentum has been increasingly recognized not only as a peritoneal extension for visceral protection but also as a highly dynamic immunometabolic organ that actively participates in immune surveillance [1]. Its rich vascularization, extensive lymphatic network, and high immune cell density make it an important abdominal cavity defense site, acting in antigen capture, leukocyte recruitment, and the containment of infectious and inflammatory processes [2,3]. It may, however, also favor the persistence of infectious agents and inflammation chronicity [4].

This functional duality makes the omentum particularly relevant in canine leishmaniosis (CanL), a zoonosis of major One Health importance caused by *Leishmania infantum*, with dogs comprising the main urban reservoir in endemic areas. This infection exhibits tropism for mononuclear phagocyte system cells, particularly macrophages, triggering a complex inflammatory response, as well as multisystemic manifestations ranging from subclinical forms to severe conditions involving cutaneous, hematological, and visceral impairment [5,6,7].

Studies on CanL immunopathogenesis typically focus on classical lymphoid and visceral organs, such as the spleen, liver, bone marrow, and lymph nodes, in which the presence of the parasite and inflammatory alterations have been widely described. However, increasing evidence has demonstrated that *L. infantum* exhibits a broad capacity for tissue dissemination, reaching still poorly explored extranodal compartments, including the peritoneal cavity, cavitary fluids, and visceral adipose tissues [8,9,10,11]. These findings expand the understanding of infection dynamics, indicating that CanL may involve distinct microenvironments beyond classically recognized sites.

In this context, the omentum role in CanL immunopathogenesis is still poorly understood, with scarce studies investigating omental inflammatory responses in naturally infected dogs and rare reports of amastigote forms in this tissue. Considering its unique immunological composition and its close anatomical relationship with the abdominal cavity, the omentum may represent not only a secondary inflammatory involvement site but also a potential immunologically active compartment associated with parasite persistence and chronic inflammation.

Given the above, this study aimed to assess potential omentum involvement in the immune-inflammatory response of dogs naturally infected with *L. infantum* through histopathological tissue characterizations, amastigote form identification and systemic inflammatory biomarker correlations.

## 2. Material and Methods

A total of 40 adult male and female mixed-breed dogs between 1 and 14 years old and weighing up to 35 kg were investigated. All animals underwent complete clinical examinations followed by serological CanL testing, including the rapid immunochromatographic test DPP^®^ LCan (Bio-Manguinhos^®^, Rio de Janeiro, RJ, Brazil) and an enzyme-linked immunosorbent assay (ELISA), according to Brazilian Ministry of Health recommendations [12].

Following clinical and serological evaluations, animals were classified into two study groups, namely a control group, consisting of 10 asymptomatic seronegative dogs, and a symptomatic group, comprising 30 dogs naturally infected with *L. infantum* and presenting clinical manifestations compatible with CanL. Clinical manifestations were recorded as binary variables (absent = 0, present = 1), including weight loss, cachexia, skin lesions, keratoconjunctivitis, ophthalmopathy, onychogryphosis, lymphadenomegaly, hepatomegaly, splenomegaly, ascites, and seizures.

Obese animals and those with evident neoplasms, diagnosed coinfections, systemic septic processes, abdominal inflammatory diseases unrelated to leishmaniosis, and severe macroscopic alterations that could compromise histopathological omentum interpretations were excluded from the study. Vector-borne coinfections were screened using the SNAP^®^ 4Dx^®^ Plus Test (IDEXX Laboratories, Westbrook, ME, USA). Additionally, control dogs were required to be seronegative, clinically healthy, and free of abdominal inflammatory lesions upon surgical inspection.

Complete blood counts and serum biochemical analyses were performed by the same reference laboratory. Hematological parameters were determined employing an automated veterinary hematology analyzer (BC-2800Vet, Mindray^®^, Shenzhen, China), with microscopic blood smear evaluations and biochemical analyses that included serum urea, creatinine, alkaline phosphatase, aspartate aminotransferase, alanine aminotransferase, total protein, albumin, globulins, and bilirubin levels. These data were subsequently used to calculate neutrophil-to-lymphocyte ratios (NLR), neutrophil-to-albumin ratios (NAR), albumin-to-globulin ratios (AGR), platelet-to-lymphocyte ratios (PLR), and monocyte-to-lymphocyte ratios (MLR), all of which were investigated as systemic inflammatory biomarkers and correlated with microscopic omental findings.

Dogs belonging to the symptomatic group were subjected to pre-anesthetic medication employing acepromazine maleate (0.05 mg/kg), xylazine hydrochloride (1 mg/kg), and ketamine hydrochloride (10 mg/kg), administered intramuscularly. Following anesthetic plane confirmation, absence of nociceptive reflexes, and complete loss of consciousness, a 19.1% potassium chloride solution (1 mL/kg) was administered intravenously to induce cardiorespiratory arrest, according to humane euthanasia recommendations [13].

Following death confirmation, median celiotomies were performed for abdominal cavity and omentum inspections, evaluating visceral adipose tissue color, vascularization degree, potential tissue thickening and adhesions, friability, and any alterations suggestive of chronic inflammatory processes, such as opacity, fibrosis, and tissue retraction. Four omental fragments were then collected from each animal, measuring approximately 4 cm^2^, prioritizing areas free of traumatic or surgical artifacts. Regarding control animals, samples were opportunistically obtained during elective surgical procedures involving abdominal cavity access, with prior informed owner consent.

All tissue fragments were washed in phosphate-buffered saline and fixed in 10% buffered formalin for 24 to 48 h. Following fixation, the samples were routinely processed for histopathological assessments, by embedding in paraffin, sectioning into 2 µm thick sections, and staining with hematoxylin and eosin (HE).

Lesions were evaluated regarding chronic inflammation, vasodilation, fibrosis, granuloma formation, and intralesional amastigote *Leishmania* spp. forms. When present, inflammatory infiltrates were further categorized according to their predominant cellular composition and tissue distribution. Histological change severity was assessed applying a semiquantitative scoring system adapted from Allenspach et al. [14], according to a four-point scale (0–3), corresponding to absent, mild, moderate, and severe lesions, respectively. The final score for each parameter was calculated as the means of independent evaluations performed by two veterinary pathologists under a double-blind protocol.

All statistical analyses were performed using the Python 3.11 software (SciPy 1.13, statsmodels 0.14, and scikit-learn 1.5), with reproduction in RStudio (2026.07.0). Continuous variable normality was assessed by the Shapiro–Wilk test, and variance homogeneity was evaluated by Levene’s test based on data medians (Brown–Forsythe). Parametric variables were compared using Student’s *t*-test or Welch’s *t*-test, according to variance homogeneity, while non-parametric variables were compared using the Mann–Whitney U test. Results were expressed as means ± standard deviations or medians, with effect sizes calculated using Cohen’s d or Cliff’s δ, as appropriate.

Histological scores were analyzed by the Kruskal–Wallis test, followed by Dunn’s post hoc test with Bonferroni correction for pairwise comparisons, and the Jonckheere–Terpstra test for monotonic trend evaluations. Correlations between ordinal and quantitative variables were assessed using Spearman’s correlation coefficient (ρ). Categorical variables were compared using Fisher’s exact test or Pearson’s chi-square test, with Cramér’s V as a measure of effect size, in addition to a simple correspondence analysis for visualization.

The discriminatory capacity of the investigated inflammatory biomarkers was assessed by ROC curves, calculating the area under the curve (AUC) with 95% confidence intervals, and determinations of the optimal cutoff point using Youden’s index. Corrections for multiple comparisons were performed using the Benjamini–Hochberg method, adopting a significance level of 5% (α = 0.05).

Given the exploratory nature of this study and the relatively small sample size employed, particularly the control group, all statistical analyses were interpreted cautiously, with emphasis on effect sizes, confidence intervals, and correction for multiple comparisons rather than on isolated *p*-values.

## 3. Results

Dermatological manifestations were the most predominant alterations in CanL-positive dogs, with exfoliative lesions (90%), onychogryphosis (76.7%), and ulcerative lesions (56.7%) being the most frequent. The main systemic and visceral findings were splenomegaly (73.3%), weight loss (63.3%), lymphadenomegaly (50%), cachexia (36.7%), and hepatomegaly (23.3%). Ocular alterations were also expressive, with ophthalmopathies identified in 43.3% of the animals and keratoconjunctivitis, in 40%. Less frequent manifestations included ascites, hyperkeratosis, and seizure episodes, each observed in 3.3% of the cases.

Regarding systemic alterations, CanL-positive dogs presented hematobiochemical profiles compatible with normocytic normochromic anemia and dysproteinemia associated with hypoalbuminemia and hyperglobulinemia (Figure 1 and Figure 2). In addition, statistically significant reductions in AGR associated with increased NAR values were observed, with both inflammatory biomarkers displaying the greatest discriminatory power between groups, respectively (Figure 2 and Figure A1 and Table 1).

Macroscopically, increased vascularization, mild tissue thickening, and focal opacity areas were more frequent in the omentum of CanL-positive dogs. Mild adhesions to adjacent structures and greater friability were also observed in some cases. In control animals, the omentum was uniform in color, with preserved vascularization and no relevant macroscopic alterations.

Microscopically, preserved fibro-adipose architecture and rich vascularization were observed in the omentum of CanL-positive dogs, although chronic inflammatory alterations were observed in 86.7% of the cases, mainly characterized by diffuse lymphoplasmacytic infiltrate (53.3%), followed by perivascular (23.3%) and focal (13.3%) patterns. Granulomatous infiltrates characterized by the presence of reactive macrophages and epithelioid cells were noted in 10% of the samples. Hyperemia and interstitial fibrosis were identified in 40% and 26.7% of the cases, respectively. Amastigote *Leishmania* spp. forms were identified in 13.3% of the samples within the macrophage cytoplasm, mainly in areas associated with granulomatous infiltrates (Figure 3).

Chronic omentum inflammation degrees differed substantially between groups (δ = −0.46, *p* = 0.023), observed in only 40% of the control animals and categorized as mild, whereas CanL-positive dogs displayed a higher frequency of omental inflammation and more advanced involvement degrees, demonstrating greater tissue inflammatory impairment associated with infection.

The association between histological grade and type of inflammatory infiltrate was strongly demonstrated by Cramér’s V test (0.767) and Spearman’s correlation test (ρ = 0.901; *p* < 0.001), indicating an ordered inflammatory response progression. Animals presenting mild inflammation presented exclusively lymphoplasmacytic infiltrates. In moderate cases, this pattern was maintained in 71%, whereas 29% presented reactive macrophages, suggesting innate immunity recruitment. Finally, 60% of animals presenting severe inflammation exhibited granulomatous infiltrates, evidencing chronicity and greater local inflammatory response organization (Figure 4).

The inflammatory biomarkers investigated herein displayed good discriminatory capacity between groups (Figure 2), albeit with no consistent stratification among chronic omental inflammation grades (Figure 5). In contrast, the assessed erythrogram variables displayed a more consistent monotonic trend, progressively decreasing with increasing inflammatory grade (Figure 6). This indicates that omental inflammatory involvement is more strongly associated with systemic anemia severity than with classical inflammatory ratios.

Clinically relevant associations were observed between omentum systemic alterations, inflammatory biomarkers, and histological involvement in CanL-positive dogs (Figure A2). A strong negative correlation was observed between globulins and AGR (ρ = −0.78), as well as between lymphocytes and both NLR (ρ = −0.82) and PLR (ρ = −0.85), reinforcing lymphopenia and dysproteinemia systemic inflammatory response effects. Complementarily, a strong positive correlation was observed between albumin and AGR (ρ = +0.78), in addition to high concordance among erythrogram variables (ρ > 0.94), evidencing anemic profile consistency associated with CanL.

Regarding histological alterations, a higher degree of omental fibrosis was associated with reduced red blood cell and platelet counts (ρ = −0.37), whereas greater vasodilation was linked to decreased globulin and total protein levels (ρ = −0.35). A trend toward increased ALT levels was also observed with increasing inflammatory grade (ρ = +0.15), suggesting potential repercussions on other abdominal organs. Additionally, greater clinical severity was typically associated with increased NLR (ρ = +0.38), MLR (ρ = +0.36), and PLR (ρ = +0.35), indicating that clinical CanL progression is accompanied by intensified systemic inflammatory responses and greater omental microenvironment impairment.

## 4. Discussion

The clinical and laboratory findings reported herein reinforce the systemic, progressive, and inflammatory nature of CanL, particularly in symptomatic dogs naturally infected with *L. infantum*. This clinical profile is consistent with that described in the literature for dogs with symptomatic CanL and reflects broad tissue parasite dissemination, as well as persistent mononuclear phagocyte system activation [5,15,16].

The high splenomegaly and lymphadenomegaly frequencies observed in the present study may be attributed to persistent antigenic stimulation and the consequent reactive hyperplasia of lymphoid organs, whereas dermatological and ophthalmic alterations often result from immune complex deposition, vasculitis, and chronic inflammatory infiltrates, which are classically involved in CanL immunopathogenesis [17].

From a hematological perspective, normocytic normochromic anemia was observed, representing one of the most consistent CanL laboratory abnormalities and frequently associated with chronic inflammatory disease. This results from the persistent production of pro-inflammatory cytokines, reduced bone marrow due to erythropoietin, iron sequestration within the mononuclear phagocyte system, and increased erythrophagocytosis by activated macrophages [17,18]. In more advanced cases, renal impairment may also contribute to anemia progression by reducing erythropoietin production and exacerbating systemic deterioration [15].

Additionally, dysproteinemia associated with hypoalbuminemia and hyperglobulinemia represents one of the most characteristic CanL findings. Reduced albumin levels are frequently associated with the negative acute-phase response, hepatic alterations, renal losses secondary to immune-mediated glomerulopathy, and chronic nutritional impairment, whereas hyperglobulinemia results from persistent antigenic stimulation and an intense humoral immune response [5,15,17], directly contributing to decreased AGR.

Among the investigated systemic inflammatory biomarkers, AGR demonstrated the best diagnostic performance. A similar finding was described by Araújo Júnior et al. [11] in an advanced case of CanL associated with ascites and presumed monoclonal gammopathy, in which a marked AGR reduction was accompanied by an intense inflammatory response and the presence of parasitized cells in peritoneal fluid, reinforcing its value as an indicator of greater systemic impairment in CanL cases.

Furthermore, NAR is also noted as displaying relevant discriminatory capacity, as this parameter integrates cellular inflammatory responses with the metabolic-protein alterations associated with CanL. Unlike NLR, the inclusion of albumin increases its clinical sensitivity as an acute-phase response and systemic severity marker. Furthermore, the relative increase in neutrophils during initial *Leishmania* responses may reflect not only systemic inflammation but also parasite persistence and inflammatory process maintenance [11,19,20]. Thus, the superior NAR performance verified herein reinforces its potential as a complementary biomarker in clinical CanL assessments.

In contrast, NLR, PLR and MLR, although frequently associated with systemic inflammation and clinical severity in CanL cases [21], exhibited lower discriminatory capacity in the omental inflammation assessment carried out herein, potentially due to the chronic and persistent pathophysiology of the disease. Conversely, in diseases characterized by exacerbated acute inflammatory responses, such as parvovirosis associated with systemic inflammatory response syndrome, both PLR and platelet-to-neutrophil ratios (PNR) display better performance due to intense leukocytic and platelet responses [22].

In this context, immunoprotein biomarkers demonstrate greater sensitivity in the monitoring of more complex inflammatory microenvironments, like the omentum. In addition, hematological ratios derived from leukograms may be more strongly influenced by individual variables, such as age and physiological condition, requiring well-established reference intervals and careful interpretations to avoid misleading clinical conclusions [23].

The progression from mild lymphoplasmacytic omentum infiltrates to more organized granulomatous lesions demonstrates that this structure actively participates in immune-inflammatory responses in CanL cases. The predominance of mononuclear cells, especially lymphocytes, plasma cells, and macrophages, is consistent with persistent chronic inflammatory processes and reflects antigenic stimulation maintenance in response to parasite persistence, a pattern previously described in visceral organs affected by CanL [5,15,16].

The diffuse and perivascular distribution of inflammatory infiltrates demonstrates a close association between local immune activity and rich omentum vascularization, favoring leukocyte recruitment and inflammatory microenvironment maintenance. This is particularly relevant, as the omentum exhibits an extensive vascular and lymphatic network, in addition to a high concentration of resident immune cells, acting as an important immune surveillance site within the abdominal cavity [1,24].

The presence of hyperemia and fibrosis indicates that omental tissue activation is not limited to a transient event but rather progresses to progressive structural alterations. While hyperemia reflects increased blood flow and continuous cellular recruitment, fibrosis represents tissue remodeling secondary to sustained chronic inflammation, similar to that observed in other visceral adipose tissues subjected to prolonged inflammatory stimulation [2].

In more advanced stages, granuloma formation and the presence of reactive macrophages represent classical containment mechanisms against the persistence of intracellular agents. In CanL, granulomatous organization is associated with the host’s attempt to limit parasite dissemination and modulate local immune responses, especially in microenvironments rich in mononuclear phagocyte system cells [25].

The presence of *Leishmania* spp. amastigote forms within the cytoplasm of omental macrophages is highly relevant, confirming direct omentum parasitism and expanding the understanding of parasite dissemination to extranodal and cavitary sites. A similar finding was described by Araújo Júnior et al. [11], who reported the presence of amastigotes in ascitic fluid macrophages and neutrophils, reinforcing that the peritoneal cavity actively participates in CanL immunopathogenesis. The identification of amastigotes in the omentum supports the hypothesis that visceral adipose tissue may act as a functionally active immunological compartment, capable of contributing to parasite persistence and chronic inflammation maintenance. This expands the understanding of the biology of infection and reinforces the need to investigate extranodal compartments as potential parasitic reservoirs and complementary targets in prognostic assessments of the disease.

Furthermore, the association between the intensity of omental inflammation and progressive erythrocytes, hematocrit, and hemoglobin reductions suggests that microscopic omental involvement is accompanied by systemic hematological alterations rather than representing an isolated finding. As the local inflammatory response becomes more intense and organized, a progressive worsening of erythrogram variables is observed, with no corresponding association with overall clinical score. This reinforces an association between inflammatory omental involvement and the inflammatory anemia observed in CanL, comprising an important complementary clinical severity and disease prognosis indicator.

Similarly, a higher degree of fibrosis was associated with decreased red blood cell and platelet counts, while more pronounced vasodilation was associated with reduced globulin and total protein levels, indicating that microscopic omental alterations are associated with progressive hematological and biochemical changes. This supports the hypothesis that omental inflammation is an integrated component of CanL immunopathogenesis, associated with parasite persistence and systemic hematological and inflammatory alterations.

Although this study provides consistent evidence regarding omentum involvement in CanL immunopathogenesis, some methodological limitations should be considered when interpreting the findings. First, the identification of amastigote *Leishmania* spp. forms was based exclusively on HE-stained sections. The absence of complementary confirmatory techniques, such as immunohistochemistry or molecular assays, limits parasite identity, tissue parasite burden, viability, and active replication confirmations.

In addition, the relatively small sample size employed herein, particularly of the control group, should be considered when interpreting subgroup comparisons and correlation analyses. Because of this, some findings should be regarded as exploratory and require confirmation by larger, independent cohorts. Future studies combining complementary diagnostic techniques with larger study populations are, therefore, paramount to validate the presence of *Leishmania* in omental tissue and further clarify the biological significance of omental involvement in CanL.

## 5. Conclusions

This study demonstrates that the omentum actively participates in CanL immunopathogenesis. The high frequency of chronic lymphoplasmacytic infiltrates, the progression to granulomatous patterns with epithelioid macrophages, and the identification of amastigote *Leishmania* spp. forms within the macrophage cytoplasm indicate tissue parasitism and support the role of the omentum in infection chronicity and local inflammation maintenance. Furthermore, the association between histological inflammation grade and progressive erythrogram variable reduction indicates that omental involvement is associated with the intensity of systemic repercussions of the disease. Among the evaluated biomarkers, AGR is noteworthy as the main discriminatory marker, highlighting its important complementary clinical potential. These findings expand the understanding of non-classical tissues involved in *Leishmania infantum* infection and support the omentum as a relevant immunological compartment involved in inflammatory responses associated with CanL.

## Figures and Tables

**Figure 1:**
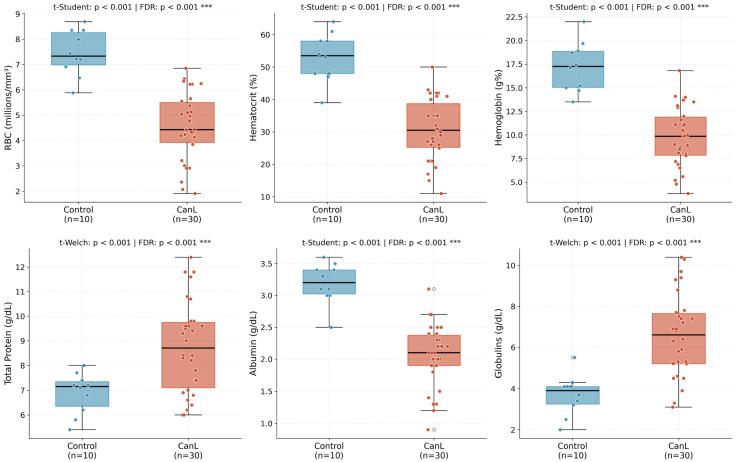
Main hematological and biochemical parameter comparisons between control from the group and dogs naturally infected with *Leishmania infantum*. *** *p* < 0.001.

**Figure 2:**
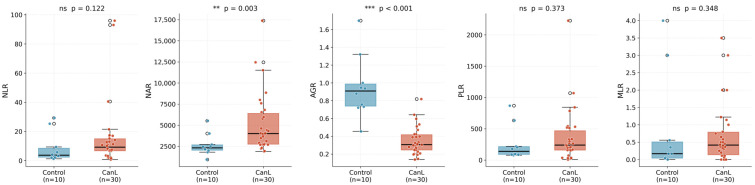
Systemic inflammatory biomarker comparisons between control dogs and dogs naturally infected with *Leishmania infantum*. NLR: neutrophil-to-lymphocyte ratio; NAR: neutrophil-to-albumin ratio; AGR: albumin-to-globulin ratio; PLR: platelet-to-lymphocyte ratio; MLR: monocyte-to-lymphocyte ratio. *** *p* < 0.001; ** *p* < 0.01; ns = not significant.

**Figure 3:**
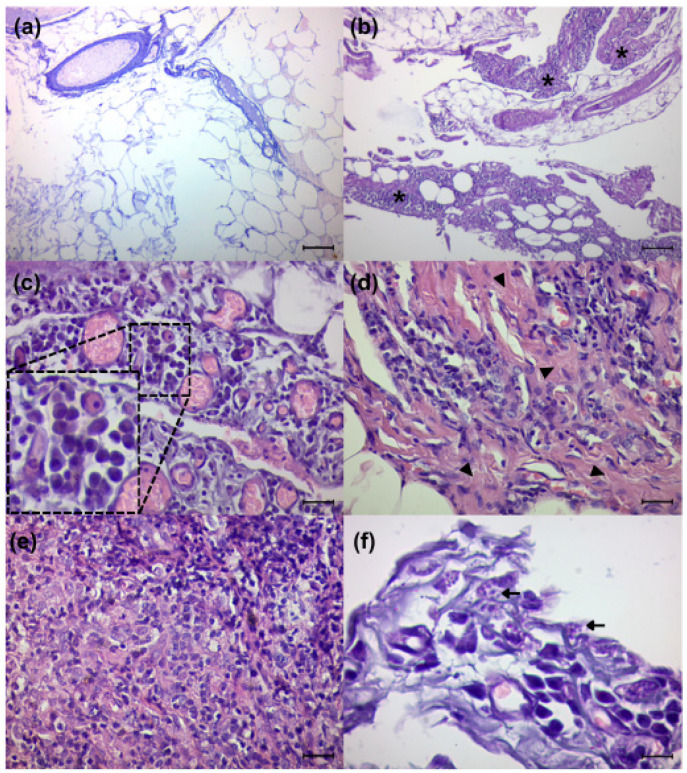
Histopathological omentum aspects in dogs naturally infected with *Leishmania infantum*. (**a**) Seronegative control animal depicting preserved histological architecture and absence of significant inflammatory changes. (**b**) Chronic mononuclear inflammatory infiltrate distributed among adipocytes (asterisks). (**c**) Lymphoplasmacytic infiltrate in the perivascular region. (**d**) Inflammatory infiltrate associated with interstitial fibrosis (arrowheads). (**e**) Granulomatous infiltrate with the presence of epithelioid macrophages. (**f**) *Leishmania* spp. amastigote forms in the macrophage cytoplasm (arrows). HE. (**a**,**b**): 100×; (**c**–**e**): 400×; (**f**): 1000×.

**Figure 4:**
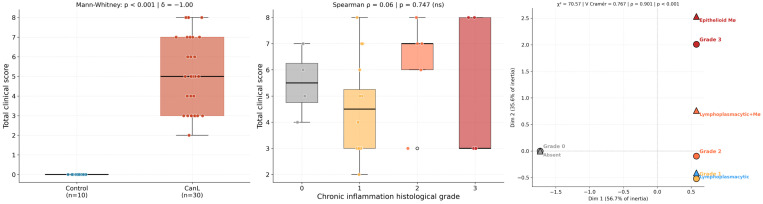
Association between clinical signs and omental inflammatory response progression of dogs naturally infected with *Leishmania infantum*. The left panel compares the total clinical score between the control and infected groups, confirming the symptomatic profile of the experimental animals. The central panel depicts the relationship between total clinical score and histological chronic omental inflammation grade, indicating no significant correlation between clinical severity and inflammatory grade. The right panel presents the correspondence analysis between histological grade and the predominant inflammatory infiltrate. Dim 1: separates animals without inflammatory lesions from those presenting any degree of omental inflammation; Dim 2: orders the inflammatory infiltrates according to increasing biological complexity, ranging from a lymphoplasmacytic pattern to epithelioid macrophages. Together, both dimensions explain 92.3% of the total inertia, indicating a clear association between inflammatory severity and infiltrate composition changes.

**Figure 5:**
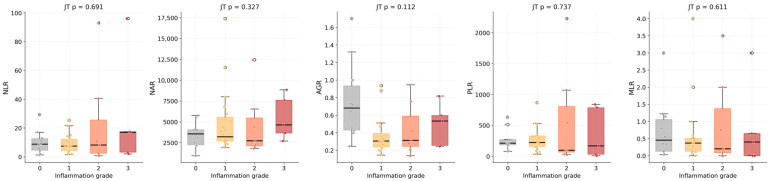
Association between systemic inflammatory biomarkers and histological chronic inflammation grade in the omentum of dogs naturally infected with *Leishmania infantum*. NLR: neutrophil-to-lymphocyte ratio; NAR: neutrophil-to-albumin ratio; AGR: albumin-to-globulin ratio; PLR: platelet-to-lymphocyte ratio; MLR: monocyte-to-lymphocyte ratio. JT: Jonckheere–Terpstra.

**Figure 6:**
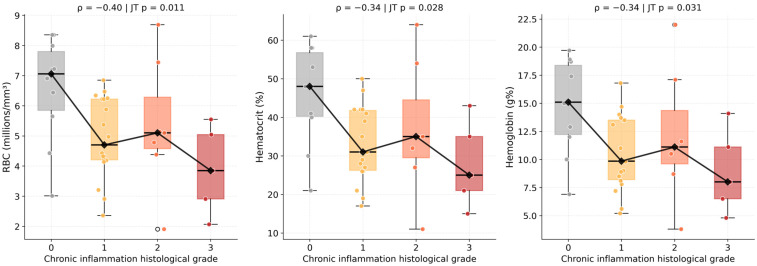
Association between histological chronic omental inflammation grade and erythrogram variables in dogs naturally infected with *Leishmania infantum*. The lines connecting the medians highlight the inverse relationship between histological grade and the three erythrogram variables. RBC: red blood cell; JT: Jonckheere–Terpstra.

**Table 1:** Diagnostic performance of systemic inflammatory biomarkers in control dogs and dogs naturally infected with *Leishmania infantum*.

Biomarker	Cutoff	95% CI	Sensitivity	Specificity	AUC
NLR	>6.73	[0.483; 0.850]	76.7%	70.0%	0.667
NAR	>2730	[0.681; 0.952]	80.0%	80.0%	0.817
AGR	<0.64	[0.922; 1.000]	96.7%	90.0%	0.970
PLR	>230.88	[0.399; 0.794]	53.3%	80.0%	0.597
MLR	>0.20	[0.405; 0.799]	73.3%	60.0%	0.602

95% CI calculated using the Hanley-McNeil method. NLR: neutrophil-to-lymphocyte ratio; NAR: neutrophil-to-albumin ratio; AGR: albumin-to-globulin ratio; PLR: platelet-to-lymphocyte ratio; MLR: monocyte-to-lymphocyte ratio; AUC: area under the curve; >indicates values above the cutoff; <indicates values below the cutoff.

**Figure A1:**
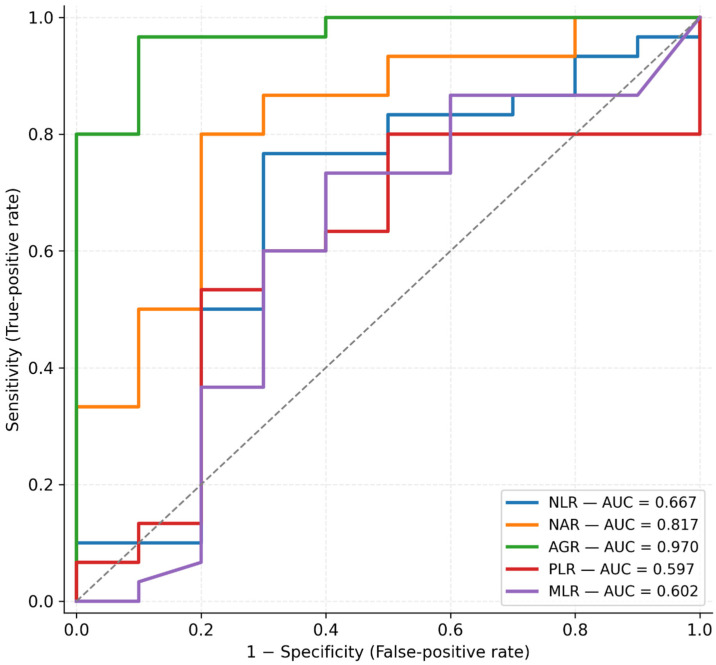
Receiver operating characteristic (ROC) curves of systemic inflammatory biomarkers used to discriminate between control dogs and dogs naturally infected with *Leishmania infantum*. AUC: area under the curve; NLR: neutrophil-to-lymphocyte ratio; NAR: neutrophil-to-albumin ratio; AGR: albumin-to-globulin ratio; PLR: platelet-to-lymphocyte ratio; MLR: monocyte-to-lymphocyte ratio.

**Figure A2:**
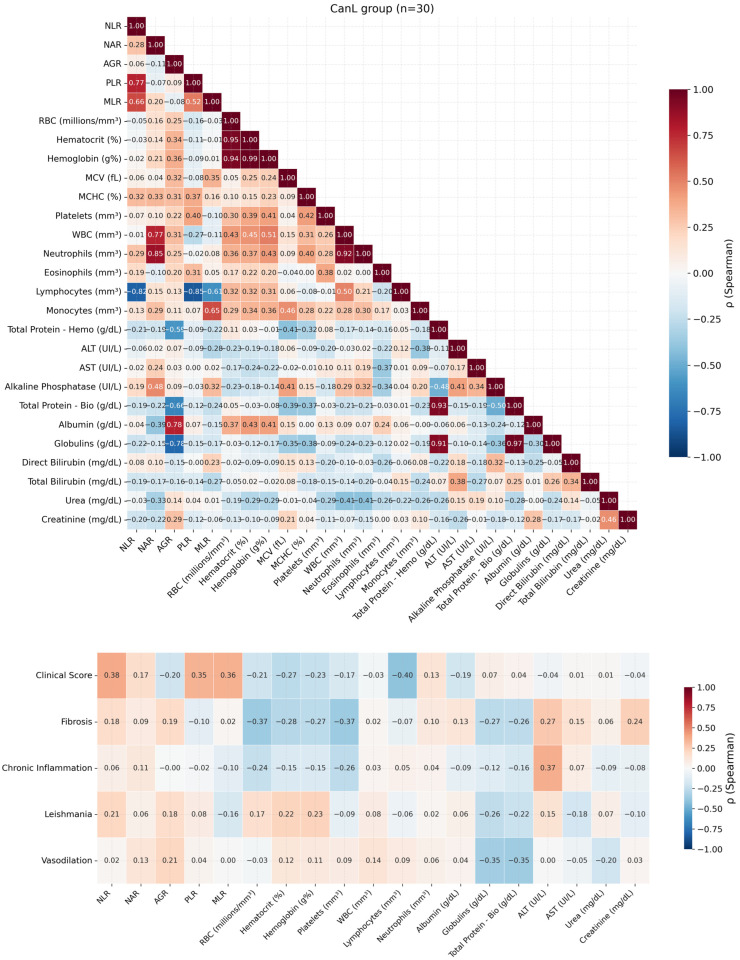
Heatmaps representing Spearman’s correlation between hematological parameters, biochemical parameters, systemic inflammatory biomarkers, and microscopic alterations in the omentum of dogs naturally infected with *Leishmania infantum*. In the upper portion of the figure, the correlation between hematobiochemical variables and systemic inflammatory biomarkers (NLR, NAR, AGR, PLR, and MLR) is shown. In the lower portion of the figure, the correlation between the animals’ clinical score, hematobiochemical tests, inflammatory biomarkers, and omental histopathological findings is presented. The intensity and direction of the correlations are represented by the color scale, ranging from −1 (perfect negative correlation) to +1 (perfect positive correlation), according to Spearman’s correlation coefficient (ρ). AGR: albumin-to-globulin ratio; ALT: alanine aminotransferase; AST: aspartate aminotransferase; MCHC: mean corpuscular hemoglobin concentration; MCV: mean corpuscular volume; MLR: monocyte-to-lymphocyte ratio; NAR: neutrophil-to-albumin ratio; NLR: neutrophil-to-lymphocyte ratio; PLR: platelet-to-lymphocyte ratio; RBC: red blood cells; WBC: white blood cell.

## Data Availability

The original contributions presented in this study are included in the article. Further inquiries can be directed to the corresponding authors.

## Abbreviations

The following abbreviations are used in this manuscript:

AGR	Albumin-to-globulin ratio
ALT	Alanine aminotransferase
AST	Aspartate aminotransferase
AUC	Area under the curve
CanL	Canine leishmaniosis
CI	Confidence interval
HE	Hematoxylin and eosin
JT	Jonckheere–Terpstra
KW	Kruskal–Wallis
MCHC	Mean corpuscular hemoglobin concentration
MLR	Monocyte-to-lymphocyte ratio
MCV	Mean corpuscular volume
NAR	Neutrophil-to-albumin ratio
NLR	Neutrophil-to-lymphocyte ratio
PLR	Platelet-to-lymphocyte ratio
PNR	Platelet-to-neutrophil ratio
RBC	Red blood cell
ROC	Receiver operating characteristic
WBC	White blood cell

## References

[1] Huyghe S., de Rooster H., Doom M., van den Broeck W. (2015). The microscopic structure of the omentum in healthy dogs: The mystery unravelled. Anat. Histol. Embryol..

[2] Wang A.W., Prieto J.M., Cauvi D.M., Bickler S.W., de Maio A. (2020). The greater omentum—A vibrant and enigmatic immunologic organ involved in injury and infection resolution. Shock.

[3] Liu M., Silva-Sanchez A., Randall T.D., Meza-Perez S. (2020). Specialized immune responses in the peritoneal cavity and omentum. J. Leukoc. Biol..

[4] Fedorchenko I., Maksymenko O., Hryn V., Bilash V., Tykhonova O., Tarasenko Y. (2025). The greater omentum as a peripheral organ of the immune system: Morphological and functional aspects. East. Ukr. Med. J..

[5] Baneth G., Koutinas A.F., Solano-Gallego L., Bourdeau P., Ferrer L. (2008). Canine leishmaniosis—New concepts and insights on an expanding zoonosis: Part one. Trends Parasitol..

[6] Kaye P., Scott P. (2011). Leishmaniasis: Complexity at the host—Pathogen interface. Nat. Rev. Microbiol..

[7] Vilas-Boas D.F., Nakasone E.K.N., Gonçalves A.A.M., Lair D.F., Oliveira D.S.d., Pereira D.F.S., Silva G.G., Conrado I.d.S.S., Resende L.A., Zaldívar M.F. (2024). Global distribution of canine visceral leishmaniasis and the role of the dog in the epidemiology of the disease. Pathogens.

[8] da Silva A.V.M., de Paula A.A., Cabrera M.A.A., Carreira J.C.A. (2005). Leishmaniose em cães domésticos: Aspectos epidemiológicos. Cad. Saude Publica.

[9] Dantas-Torres F. (2006). Presence of Leishmania amastigotes in peritoneal fluid of a dog with leishmaniasis from Alagoas, Northeast Brazil. Rev. Inst. Med. Trop. Sao Paulo.

[10] Brito M.C.P., Sousa M.d.F., Sampaio R.A.G., Linhares M.T., Riquelme L.F., Lacerda W.K., Lucena R.B. (2024). Canine leishmaniosis associated with acute pleural effusion and sudden death in a dog. Vet. Sci..

[11] Araújo-Júnior H.N., Frota J.V.S., Ferreira T.C., Nunes-Pinheiro D.C.S. (2026). Presumed monoclonal gammopathy and parasitized immune cells in peritoneal fluid of a dog with canine leishmaniasis. J. Comp. Pathol..

[12] Ministry of Health, Secretariat of Health Surveillance, Department of Epidemiological Surveillance (2026). Manual de Vigilância e Controle da Leishmaniose Visceral [Manual for Surveillance and Control of Visceral Leishmaniosis].

[13] American Veterinary Medical Association (AVMA) (2020). AVMA Guidelines for the Euthanasia of Animals: 2020 Edition.

[14] Allenspach K.A., Mochel J.P., Du Y., Priestnall S.L., Moore F., Slayter M., Rodrigues A., Ackermann M., Krockenberger M., Mansell J. (2018). Correlating gastrointestinal histopathologic changes to clinical disease activity in dogs with idiopathic inflammatory bowel disease. Vet. Pathol..

[15] Solano-Gallego L., Miró G., Koutinas A., Cardoso L., Pennisi M.G., Ferrer L., Bourdeau P., Oliva G., Baneth G. (2011). LeishVet guidelines for the practical management of canine leishmaniosis. Parasites Vectors.

[16] Meléndez-Lazo A., Ordeix L., Planellas M., Pastor J., Solano-Gallego L. (2018). Clinicopathological findings in sick dogs naturally infected with *Leishmania infantum*: Comparison of five different clinical classification systems. Res. Vet. Sci..

[17] Koutinas A.F., Koutinas C.K. (2014). Pathologic mechanisms underlying the clinical findings in canine leishmaniosis due to *Leishmania infantum/chagasi*. Vet. Pathol..

[18] Nicolato R.d.C., de Abreu R.T., Roatt B.M., Aguiar-Soares R.D.d.O., Reis L.E.S., Carvalho M.d.G., Carneiro C.M., Giunchetti R.C., Bouillet L.E.M., Lemos D.S. (2013). Clinical forms of canine visceral leishmaniasis and related myelogram and hemogram changes. PLoS ONE.

[19] Peters N.C., Egen J.G., Secundino N., Debrabant A., Kimblin N., Kamhawi S., Lawyer P., Fay M.P., Germain R.N., Sacks D. (2008). In vivo imaging reveals an essential role for neutrophils in leishmaniasis transmitted by sand flies. Science.

[20] Ribeiro-Gomes F.L., Peters N.C., Debrabant A., Sacks D.L. (2012). Efficient capture of infected neutrophils by dendritic cells in the skin inhibits the early anti-Leishmania response. PLoS Pathog..

[21] Monroig M., Navarro P.F., Movilla R., Díaz B., Tabar-Rodriguez M.D., Melero Q., Ribas A., Mesa I., Castro-López J., Gil-Vicente L. (2026). Hematological ratios in Leishmania infantum–seropositive and seronegative dogs and their distribution across clinical stages. Animals.

[22] Sevim K., Çolakoğlu E.Ç., Kaya U. (2025). Evaluation of hematologic indices in parvovirus infected dogs with systemic inflammatory response syndrome (SIRS). Top. Companion Anim. Med..

[23] Navarro P.F., Monroig M., Gil-Vicente L. (2025). Reference intervals for hematological inflammatory ratios in healthy dogs. Animals.

[24] Di Nicola V. (2019). Omentum a powerful biological source in regenerative surgery. Regen. Ther..

[25] Rangel-Moreno J., Moyron-Quiroz J.E., Carragher D.M., Kusser K., Hartson L., Moquin A., Randall T.D. (2009). Omental milky spots develop in the absence of lymphoid tissue-inducer cells and support B and T cell responses to peritoneal antigens. Immunity.

